# The Adroitness of Andrographolide as a Natural Weapon Against Colorectal Cancer

**DOI:** 10.3389/fphar.2021.731492

**Published:** 2021-11-02

**Authors:** Silpita Paul, Dia Roy, Subhadip Pati, Gaurisankar Sa

**Affiliations:** Division of Molecular Medicine, Bose Institute, Kolkata, India

**Keywords:** Colorectal cancer, Andrographolide, Phytochemical, immunomodulator, Antitumor

## Abstract

The conventional carcinoma treatment generally encompasses the employment of radiotherapy, chemotherapy, surgery or use of cytotoxic drugs. However, recent advances in pharmacological research have divulged the importance of traditional treatments in cancer. The aim of the present review is to provide an overview of the importance of one such medicinal herb of Chinese and Indian origin: *Andrographis paniculate* on colorectal cancer with special emphasis on its principal bioactive component andrographolide (AGP) and its underlying mechanisms of action. AGP has long been known to possess medicinal properties. Studies led by numerous groups of researchers shed light on its molecular mechanism of action. AGP has been shown to act in a multi-faceted manner in context of colorectal cancer by targeting matrix metalloproteinase-9, Toll-like receptor or NFκB signaling pathways. In this review, we highlighted the recent studies that show that AGP can act as an effective immunomodulator by harnessing effective anti-tumor immune response. Recent studies strongly recommend further research on this compound and its analogues, especially under *in-vivo* condition to assess its actual potential as a prospective and efficient candidate against colorectal cancer. The current review deals with the roles of this phytomedicine in context of colorectal cancer and briefly describes its perspectives to emerge as an essential anti-cancer drug candidate. Finally, we also point out the drawbacks and difficulties in administration of AGP and indicate the use of nano-formulations of this phytomedicine for better therapeutic efficacy.

## Introduction

Colorectal cancer (CRC) is one of the highest occurring malignancies worldwide. In 2020, approximately 147,950 individuals were diagnosed with CRC with a 35% mortality rate. Around a 20% mortality rate has been observed in patients below 50 years of age (Siegel et al., 2020). The conventional therapies include surgery, chemotherapy, and targeted therapy. The current chemotherapeutic drugs treat CRC, but that cause adverse toxicity. It also develops drug resistance. The targeted therapy is quite promising, but sometimes it lacks specificity, is not cost-effective and might associate with adverse events (Wolpin and Mayer, 2008; Xie et al., 2020). Nowadays, phytochemicals are considered a natural arsenal to fight against cancer risk. The foremost advantage of phytochemical usage is that it can treat the malignancy with less or no adverse side effects (Ranjan et al., 2019). Andrographolide (AGP) is a phytochemical which has anti-cancer properties and many studies have reported its wide use as a weapon to fight against cancer (Mishra et al., 2015).

The natural source of AGP is the plant, *Andrographis paniculata* (Burm.f.) of the family *Acanthaceae*. This medicinal plant is widely cultivated and used in India, Sri Lanka, China and many other South Asian countries for its curative properties against several diseases and infections (Hossain et al., 2014). The extract of *A. paniculata*, i.e., AGP has several biological activities, such as antioxidant (Xu et al., 2019), antiviral, antibacterial (Wiart et al., 2005), anti-inflammatory (Abu-Ghefreh et al., 2009), antipyretic (Madav et al., 1995), anti-thrombotic, hepatoprotective (Pan et al., 2017), and most importantly, it has anti-cancer properties (Farooqi et al., 2020). AGP also acts as an immunomodulator or immunostimulant (Churiyah, 2015).

The leaves of *A. paniculata* contain several bioactive components, which includes diterpene lactones (deoxy andrographolide, andrographolide, neoandrographolide, and 14-deoxy-11, 12-didehydro andrographolide), diterpene glucoside (deoxyandrographolide 19_-d-glucoside), and flavonoids (5, 7, 20, 30-tetramethoxyflavanone and 5-hydroxy-7, 20, 30-trimethoxyflavone) (Akbar, 2011). Among them, AGP (C_20_H_30_O_5_) is the main bioactive component of the plant (Chakravarti and Chakravarti, 1951). Due to the presence of lactone diterpene, the component and the plant taste bitter. Numerous *in vitro* and *in vivo* studies previously reported the anticancer property of AGP (Rajagopal et al., 2003). In this review, we tried to accumulate various aspects of the component with a focus on the immunomodulatory and anticancer role. We also discussed its application for the treatment of CRC as an independent drug or as an adjuvant.

## The Phytochemical From the Herb *A. Paniculata* and its Analogues

**FIGURE 1:**
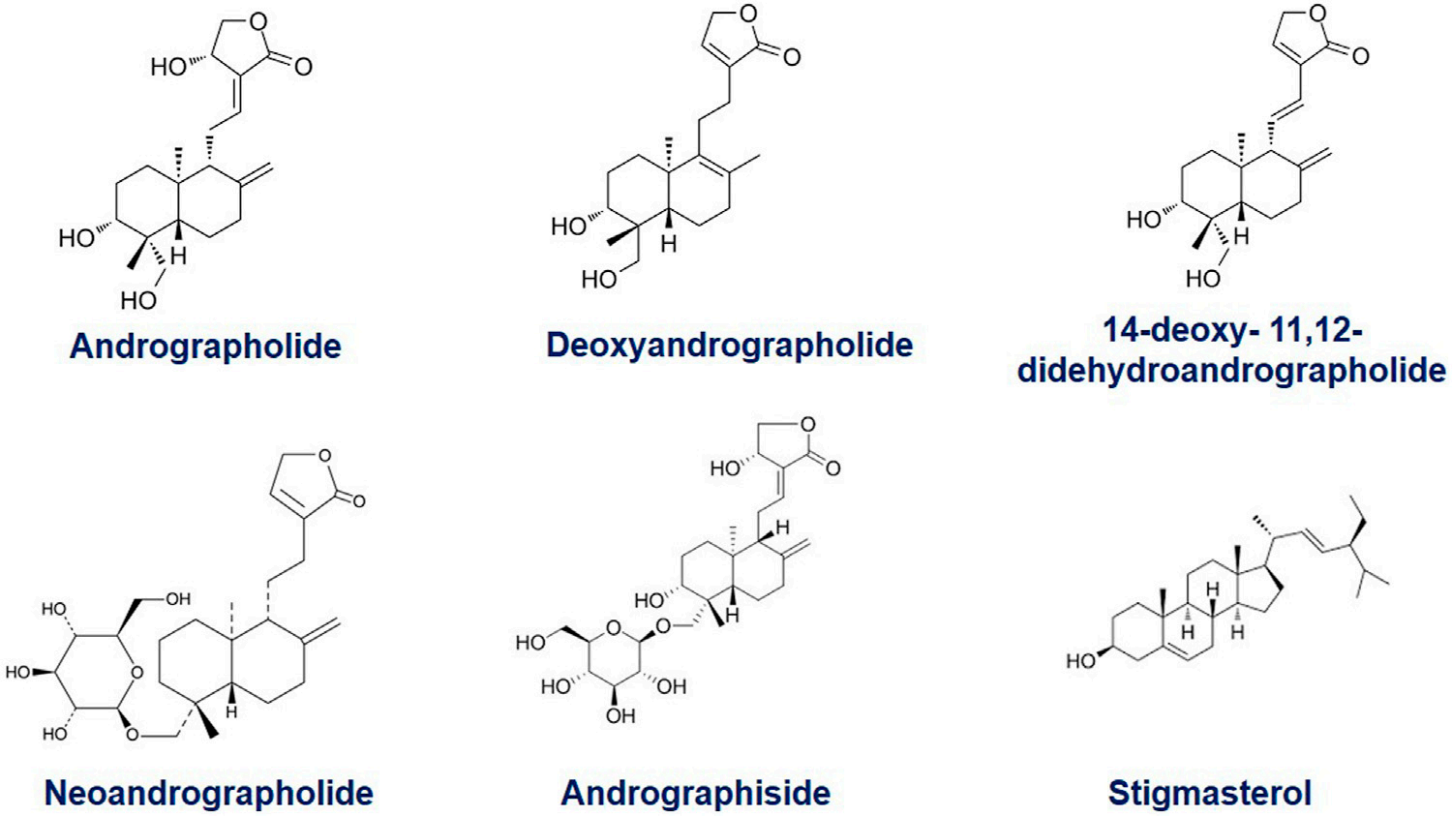
The organic structure of Andrographolide (C_20_H_30_O_5_), containing a-alkylidene c-butyrolactone moiety; two olefin bonds Δ8 (17) and Δ12 (13), and three hydroxyls at C-3, C-14, and C-19 and its common analogues, Deoxyandrographolide, 14-deoxy- 11, 12-didehydroandrographolide, Neoandrographolide, Andrographoside, Stigmasterol.

The main bioactive compound of the plant is andrographolide/AGP (C_20_H_30_O_5_), containing 1.84% of the plant extract. It is a colorless, crystal-like, extremely bitter taste compound and holds lactone (bicyclic diterpenoid lactone) functioning (Bera et al., 2014). The organic structure of AGP consists of a-alkylidene c-butyrolactone moiety, two olefin bonds Δ8 and Δ12, and three hydroxyls at C-3, C-14, and C-19. The other analogues metabolites of *A. paniculata* exhibit equal prominence in the field of phyto-pharmaceuticals. It contains several diterpenoids and diterpenoid glycosides, such as neo andrographolide, deoxy andrographolide, 14-deoxyandrographolide, 14-deoxy- 11, 12-didehydro andrographolide, andrographiside, deoxyandrographiside, homo andrographolide, andrographan, andrographon, andrographosterin, and stigmasterol. The 14-deoxy- 11, 12-didehydroandrographolide is an isolated labdane diterpenoid (Mishra et al., 2015). Several other chemical compounds of *A. paniculata* are flavonoids, and xanthones in nature (Matsuda et al., 1994; Dua et al., 2004). The extracted flavonoids are 5-years droxy-7, 8, 6, 2′, 4′-trimethoxyflavone, 5,6-Dihydroxy-7,8-dimethoxyflavone, and 5-hydroxy-7,8- dimethoxyflavone (Mishra et al., 2007). During the last several years, various researchers analyzed the chemical nature of *A. paniculata*. According to the study, 19 AGP analogues were found based on structure-activity relation. Out of them, several analogues exhibited higher cytotoxic activities than the parent compounds (Sirion et al., 2012). The functional analysis of 14-deoxy-11, 12-didehyroandrogrpholide showed a convincing grip in controlling the cell cycle process and cell cycle arrest in breast carcinoma cells. It also caused autophagy in cancer cells (Tan et al., 2012). The primary carbon structures of these compounds are presented in Figure 1.

## The Molecular Pathways Invovled in Colorectal Cancer

**FIGURE 2:**
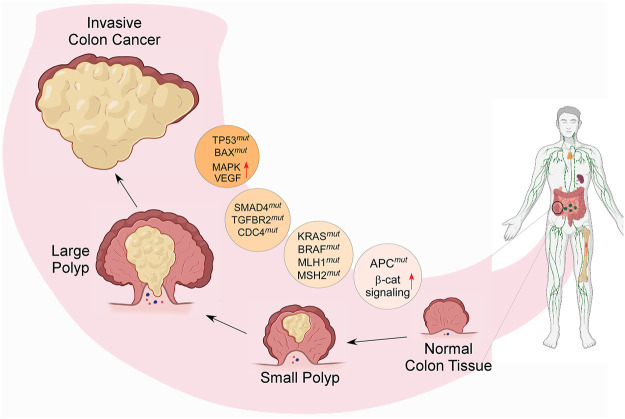
Diagram representing the molecular cause of CRC and its gradual development from small adenoma polyp to invasive cancerous form; the process is modulated by several genetic modifications, such as chromosomal instability; mutations in tumor suppressor genes and DNA mismatch repair genes, such as *TP53*, *MLH1*, *MSH2*, SMAD4, *TGFBR2*, CDC4; stimulation of oncogenic signaling pathways, such as K-RAS, BRAF, MAPK; and activation of the growth factors, such as COX2, EGF & VEGF- mediated signaling. (*β*-cat = *β*-catenin, mut = mutation).

CRC is the consequence of multiple genetic and epigenetic anomalies that occur within cells. The abnormalities of several signaling pathways are also associated with CRC. A colorectal adenoma turns into carcinoma because of chromosomal anomalies, microsatellite instability, and CpG island methylator phenotyping. Since it is a heterogeneous disease, it requires in-depth molecular understanding for the development of targeted therapies. The molecular basis of CRC involves several factors that initiate tumor formation and progression (Nguyen and Duong, 2018). This section summarizes the molecular mechanisms and signaling pathways associated with CRC (Figure 2, created by BioRender and Canvas).

### Chromosomal Instability

Chromosomal instability is one of the vital causes of CRC. In the case of CRC, genetic aberrations occur in the genes. These aberrations are responsible for maintaining chromosomal instability (Barber et al., 2008). Several changes in the chromosomal copy number (Lengauer et al., 1997) and loss of wild-type copy of the tumor-suppressor genes, such as APC, P53 cause CRC (Markowitz and Bertagnolli, 2009) in most patients.

### Defects in DNA Repair Mechanism

Inactivation of the mismatch repair genes of DNA causes CRC. In most cases, it is hereditary in nature. Germ-line mutation in mismatch repair genes, *MLH1* and *MSH2* confirm a lifetime risk of CRC in 80% cases, who suffered from hereditary nonpolyposis colon cancer (HNPCC) (Fishel et al., 1993; Bronner et al., 1994). This mismatch repairing anomaly causes genomic instability and instigates the development of CRC in HNPCC patients (Järvinen et al., 2000). Bi-allelic silencing of the promoter region of *MLH1* by methylation hampers mismatch repairing, thus causes non-familial CRC in 15% of patients. Mismatch repair defects also inactivate the tumor suppressor genes that encode *TGFBR2*, *BAX*; base excision repair gene *MYH,* which eventually leads to the CRC (Al-Tassan et al., 2002; Markowitz and Bertagnolli, 2009) in numbers of patients.

### Abnormal DNA Methylation

Epigenetic silencing of genes due to abnormal DNA methylation is another vital cause of CRC generation (Kondo and Issa, 2004). In the normal genome, cytosine methylation does not occur in CpG islands. But in the case of the CRC genome, abnormal methylation occurs within promoter-associated CpG islands (Issa, 2004). This phenomenon induces epigenetic silencing. In sporadic CRC with microsatellite instability, the aberrant methylation shuts down the *MLH1* expression (Toyota et al., 1999). The association between *MLH1* silencing and CRC is well-established, but the detailed mechanism of colorectal carcinogenesis still requires attention (Barault et al., 2008).

### Mutation of Tumor Suppressor Genes

The APC gene silencing due to mutation improperly activates the Wnt signaling pathway, which helps in developing familial adenomas polyposis and subsequently leads to the risk of CRC (Korinek et al., 1997; Goss and Groden, 2000). Mutation of the *TP53* gene is another major cause of CRC. Both copies of *TP53* alleles are knocked down either by missense mutation or by 17p chromosomal deletion that eventually impedes the cell cycle arrest of the abnormal cells, causes CRC (Markowitz and Bertagnolli, 2009). Somatic mutation of *TGFBR2* occurs in about one-third of CRC patients that causes inactivation of TGF*β* signaling (Seoane and Gomis, 2017). Missense mutations of *the TGFBR2* kinase domain inactivate the downstream TGF*β* pathway factors, SMADs, and subsequently develop CRC from adenoma (Grady et al., 1998).

### Stimulation of Oncogenic Signaling Pathways

RAS and BRAF play a significant role in developing CRC. The oncogenic mutations of these two genes activate the MAPK pathway in 37% and 13% of CRC patients (Bos et al., 1987), respectively. K-RAS mutation activates GTPase activity that further stimulates RAF. Similarly, BRAF mutations activate BRAF serine-threonine kinase activity, consequently activate MAPK signaling cascade (Rajagopalan et al., 2002). Genetic alteration of *PI3KCA*, down-regulation of PTEN, up-regulation of IRS2, AKT, and PAK4 activate PI3K signaling pathway that eventually led to the development of the CRC (Samuels et al., 2004).

### Activation of the Growth Factor Pathways

The activation of growth factor pathways helps in cancer progression. The up-regulation of COX2, activation of prostaglandin signaling, and synthesis of prostaglandin E2 are highly associated with CRC (Cha and DuBois, 2007). Several clinical trials reported that COX2 inhibition by chemotherapeutic drugs prevents the progression of new adenomas in the colorectal region and subsequently reduces the size of the formed adenomas (Arber et al., 2006). Numerous studies have reported that EGFR-mediated signaling causes CRC. EGFR activates PI3K and MAPK signaling cascades that eventually cause malignancy (Saltz et al., 2004). Another major growth factor is VEGF, which plays a significant role in the angiogenesis process. Activation of the VEGF signaling pathway is highly lethal to CRC patients (Hurwitz et al., 2004).

## Immunomodulatory Role of Andrographolide

AGP as an immunomodulator in various types of cancer has been a raging field of research for the last few decades. AGP enhances the natural killer (NK) cells activity in tumor-bearing mice (Sheeja and Kuttan, 2007a). It increases IL2 and IFN*ɣ* secretion by T cells that suppress tumor growth (Sheeja and Kuttan, 2007b). It prevents detrimental autoimmune responses by inducing antigen-specific tolerance (Iruretagoyena et al., 2006). AGP reduces the mRNA expression of inflammatory cytokines in LPS activated macrophages (Qin et al., 2006; Wang et al., 2010). HN-02 is a mixture of AGP, 14-deoxy andrographolide, and 14-deoxy-11, 12 di-dehydroandrographolide. It enhances immunological activities either by modifying the immune responses during antigen reaction or by reversing the cyclophosphamide-induced immune suppression. It stimulates the response of macrophages and the action of lymphocytes in antibody synthesis and secretion. It improves immunity by activating intricate cellular pathways to increase the efficacy of antigen-clearance by phagocytes or by inducing the secretion of immune effector molecules (Naik and Hule, 2009).

It is a well-known fact that macrophages play a vital role in shaping tumor-mediated immune response (Shapouri-Moghaddam et al., 2018). Recent studies have shown the potential effect of this phytomedicine in modulating the innate arm of the immune system by affecting macrophage phenotypic polarization (Davoodvandi et al., 2019). MAPK and PI3K signaling cascades play a role in the AGP-mediated macrophage activation and their subsequent polarization. In neutrophils, this phytomedicine blocks ROS production. The administration of AGP up-regulates the expression of CD markers and the cytokine TNF*α*. These improve the cytotoxicity of the lymphocytes in the tumor microenvironment (Naik and Hule, 2009). AGP treatment at a dose of 1 uM for 48 h, resulted in an increased proliferation of human peripheral blood mononuclear cells (PBMCs), which up-regulate IL2 production and increased immune response against a wide range of cancers, including colorectal cancer (Varma et al., 2011). Combinatorial administration with other drugs resulted in an escalated function of NK cells and TNF*α* thereby, resulting in better prognosis in patients affected with late-stage cancers of different types (Mishra et al., 2015). Apart from this, AGP also prevents unnecessary T cell exhaustion (Iruretagoyena et al., 2005). Experiments on animal models elucidated the potent role of AGP in increasing the antibody-mediated cellular toxicity, mitogen-induced bone marrow cell proliferation, increased production of IL2, and anti-tumor cytokine IFN*γ* in both tumor-bearing as well as healthy animal models (Sheeja and Kuttan, 2007a).

## Anticancer Properties of Andrographolide

**FIGURE 3:**
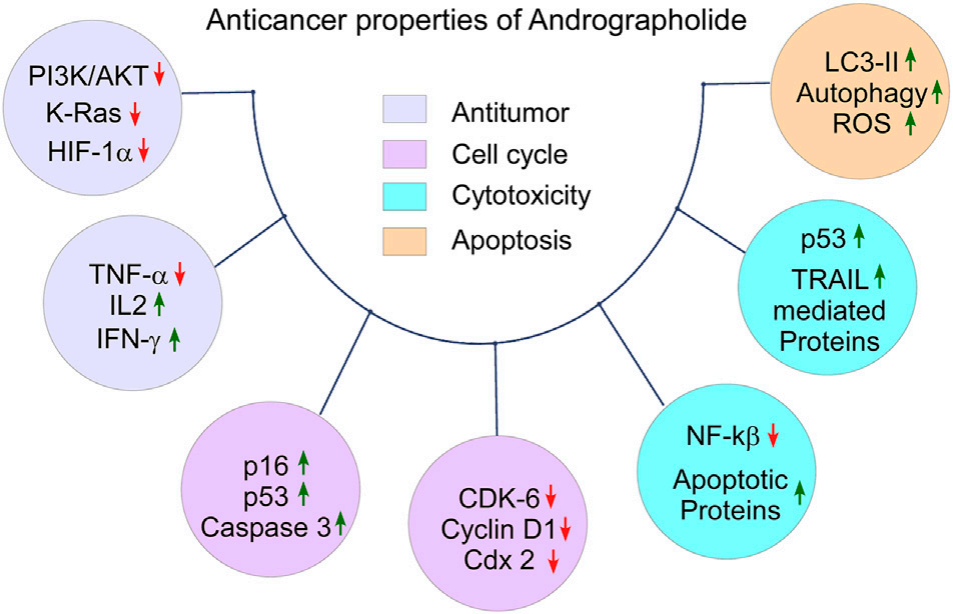
The anticancer properties of Andrographolide: Tumor suppressing property by down-regulating of tumor promoting molecules and up-regulating of anti-tumor molecules, cell cycle arrest by down-regulating of cell cycle promoting factors and up-regulating of cell cycle arresting factors in defected cells, cytotoxicity by promoting autophagy in the abnormal cell, and apoptotic property by down regulating anti-apoptotic proteins and up-regulating pro-apoptotic proteins.

The increased occurrence of cancer-associated death is a serious issue globally. The six hallmarks of cancer, i.e., proliferation, insensitivity to growth-inhibitory signals, anti-apoptosis, and induction of angiogenesis, invasion, and metastasis make cancer cell resistance against immunity. The tumor microenvironment plays a vital role in the development and progression of cancer (Hanahan and Weinberg, 2000). Phytochemicals play a significant role as anticancer agents and modulate cancer-causing pathways (Newman and Cragg, 2012). Several studies reported that AGP acts on the regulatory pathways involved in apoptosis, cell cycle, and cell adhesion process (Lai et al., 2013). Thus, AGP can be used as an anticancer agent for the treatment of cancers including CRC. AGP and its analogues can suppress the uncontrolled proliferation of cancer cell lines such as leukemia, breast, lung, and melanoma (Rajagopal et al., 2003; Kumar et al., 2004). It blocks the invasion of colon cancer cells to the distant organs (CT26 cell) (Chao et al., 2010). AGP inactivates the PI3K/AKT and ERK signaling pathways, inhibits the function of MMP2/MMP9, and suppresses the AP1 heterodimer complex. AGP inhibits cancer cell invasion and down-regulates the mRNA expression of MMP7 (Chao et al., 2010; Pratheeshkumar and Kuttan, 2011). Liu et al. reported that the molecular basis of AGP-mediated anticancer activity is associated with the inhibition of Hsp90 function and reduces the levels of Hsp90 client proteins (Liu et al., 2014). AGP and other derivatives suppress the function of the CYP1A superfamily gene, activate heterocyclic amine and carcinogenic amino acids (Jaruchotikamol et al., 2007). Although, mechanisms for the anticancer activities of AGP and its analogues may vary depending on the types of cancer cells *in vitro* and *in vivo* (Mishra et al., 2015) (Figure 3, created by BioRender and Canvas).

### Cytotoxicity

Cancer cell-specific cytotoxicity is vital for therapeutic purposes. AGP has a strong cytotoxic effect on malignant cells. AGP is cytotoxic to human hepatoma cell lines, HepG2 but has no negative effect on normal liver L-02 cells. It also exhibited cytotoxicity against human epidermoid leukemia (KB) and lymphocytic leukemia (P388) cell lines (Li et al., 2007). A study reported that AGP up regulates the autophagy markers in various cancer cell lines and induces autophagic cell death by disrupting mitochondrial membrane potential (Zhou et al., 2012). It is assumed that AGP accumulates LC3-II protein as well as autophagosomes and initiates the formation of GFP-LC3 (Chen et al., 2012). AGP and lipoic acid conjugate activate ROS-dependent DNA damage and performed cytotoxicity by inducing apoptosis in human leukemia K562 cells (Zhu et al., 2013). AGP can also act as an anti-proliferative agent. AGP can penetrate the blood-brain barrier. Therefore, in case of glioblastoma, it induces cell cycle arrest at the G2/M phase and down regulates cdk1 and cdc25 proteins. It causes cytotoxicity in glioblastoma cells by suppressing the function of the PI3/Akt signaling pathway (Li et al., 2012). It has been observed that the other AGP analogues are equally cytotoxic to malignant cells (Chen et al., 2013).

### Apoptosis Induction

Programmed cell death or apoptosis is a molecular mechanism that activates a conserved intracellular pathway and is highly antagonist with cancer progression and metastasis. Cancer cells are characteristically apoptotic resistant (Kaufmann and Earnshaw, 2000). Several studies reported that AGP and its analogues successfully induce apoptosis in malignant cells. AGP activates the caspase3 and p53, prevents NFκB activity, and accordingly initiates apoptosis in human neuroblastoma cells (Sukumari-Ramesh et al., 2011). NFκB is a transcription factor that controls cell apoptosis and proliferation (Wu and Zhou, 2009). AGP-fluorouracil (5-FU) conjugate enhances apoptosis in the human hepatocellular carcinoma (HCC) cell line by activating Bax protein, caspase-3, 8, 9, and by increasing the secretion of cytochrome c (Yang et al., 2009). AGP stimulates p53-induced transcriptional up-regulation of DR4 by processing p53 phosphorylation. Subsequently, it activates the TRAIL-mediated apoptosis process (Zhou et al., 2008). IL6 expression is necessary for cell proliferation in prostate cancer, and AGP inhibits both mRNA and protein expression of IL6 that further induces apoptotic cell death (Chun et al., 2010). An analogues of AGP inhibits DNA topoisomerase II*α*, which is a vital chemotherapeutic target for anticancer agents and thus induces apoptosis in cholangiocarcinoma (Nateewattana et al., 2013). Even though the pro-apoptotic function of AGP is proved in several cancer cell lines, yet the in-depth mechanism is still ambiguous.

### Cell Cycle Arrest

The main bioactive compound of *A. paniculata* can obstruct the cell cycle process, and this interference makes it a trustworthy anticancer agent. AGP blocks tumor growth by arresting the cell cycle at the G2/M phase and initiates caspase-mediated apoptosis (Kumar et al., 2012). AGP decreases cell cycle-associated proteins, and increases the expression of cell cycle inhibitory proteins, p16, p21, p53, and thus exhibits an anti-proliferation effect in colorectal cancer cells. Shi et al. also reported that AGP treatment arrested the human colorectal carcinoma Lovo cells in the G1 phase by p27 induction and CDK4 suppression (Shi et al., 2008). Several AGP analogues, such as 3A.1 19-tert-butyldiphenylsilyl-8, 17-epoxy AGP exhibit caspase3 activation and down-regulation of CDK6, cyclin D1, and COX-2 proteins expression, which assist in cell cycle control (Nateewattana et al., 2014).

### Antitumor

AGP and analogues are successfully established as an antineoplastic drug in cancer chemotherapy with minimum side-effect on non-malignant cells. The AGP nanoparticles are efficient as the chemotherapeutic agent that show anticancer property through cell cycle arrest and apoptosis induction in breast cancer cell line (Roy et al., 2012). In AGP-based treatment, down regulation of PI3K/AKT signaling pathway has been observed that accordingly reduce the expression of HIF-1*α*, a factor for tumor growth in non-small cell lung cancer (NSCLC) by the ubiquitin-dependent degradation (Lin et al., 2011). AGP exhibits a protective role against cyclophosphamide (CTX)-induced urothelial toxicity in Swiss albino mice. AGP suppresses the production of TNF*α*, which was up-regulated during CTX administration. Furthermore, AGP increases the levels of IL2 and IFNγ that were down-regulated by CTX treatment (Sheeja and Kuttan, 2006). AGP act as a ligand to inhibit GDP-GTP exchange by coupling to transient pockets of K-Ras; thus, it reduces the GTP loading of wild type K-Ras. It further reduces the signal transmission by oncogenic mutant K-Ras: G12V by binding to Ras protein (Hocker et al., 2013). This proved the antitumor potentiality against the function of oncogenic mutant Ras. Numerous studies have already proved that AGP and its analogues could be applied as effective anticancer agents (Kasemsuk et al., 2013). AGP application in combination with cisplatin or doxorubicin increases the cancer cell-specific cytotoxicity in neuroblastoma (Sukumari-Ramesh et al., 2011), which strengthen the role of AGP as an anticancer immune-modulator.

## Mode of Action of Andrographolide in Cancer

**FIGURE 4:**
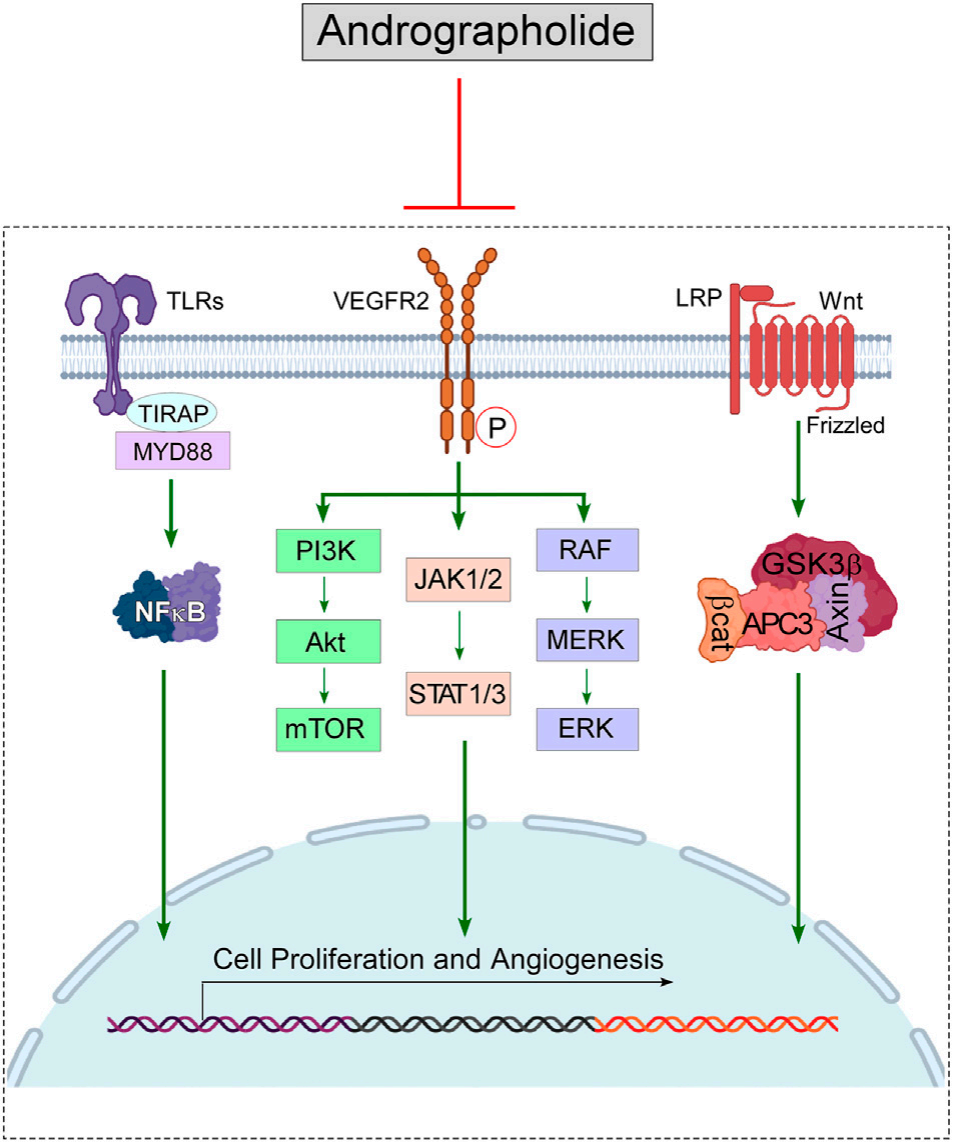
Diagram representing the immunomodulatory role of Andrographolide on the signaling pathways, that cause cancer: Andrographolide inhibits the functioning of NFκB, thus hampers the downstream signaling pathway mediated *via* MMP9; blocks the VEGFR-mediated PI3K/Akt, ERK & JAK/STAT signaling, which stimulate tumor progression; down-regulates *β*-catenine (*β*-cat) and suppresses Wnt signaling pathway to block tumor progression. Blocking of these signaling pathways lead to the suppression of cell proliferation and angiogenesis process, which eventually can cause tumor regression.

Revolutionary discovery in the arena of proteomics, as well as genomics, enabled scientists to conceptualize numerous scientific breakthroughs involving the interrelated network of tumorigenic signal events. The deregulation of cell-signaling pathways, involving, both spatial extension as well as temporal duration facilitates the tumor cells to survive under “drug-pressure” as well as enabling them to undergo epithelial-to-mesenchymal transition (EMT), metastasis, and developing an aversion to apoptosis. A special highlight is made on the efficiency of this phytochemical in modulating JAK/STAT, NFκB, Wnt/*β*-Catenin, VEGF/VEGFR, and TRAIL and mTOR-driven signaling pathways (Figure 4, created by BioRender and Canvas).

### JAK/STAT Pathway

The down-regulation of the JAK/STAT pathway is associated with cancer development. Phosphorylated STAT proteins transcriptionally up-regulate cancer-promoting genes (Haura et al., 2005). An optimum dose of AGP down-regulates IL6 at both mRNA and protein levels. It suppresses IL6-dependent autocrine loop- and paracrine loop-driven signaling by interfering with STAT3 phosphorylation (Chun et al., 2010). AGP blocks the phosphorylation of Tyr705 and Ser727 residues of STAT3 and, as well as inhibits tyrosine phosphorylation of JAK1 and JAK2 proteins (Zhou et al., 2010). Recently, STAT3 inhibitors OPB-31121 and 51602 receive clinical attention for Phase I/II clinical trials for cancer. But poor pharmacokinetic features and associated toxicities limit its usages. Hence, AGP can be used as a better replacement or an adjuvant with conventional chemical drugs as a STAT3 inhibitor (Farooqi et al., 2020).

### NFκB-Mediated Signaling

The cellular events of apoptosis and hyperplasia are closely related to the development and progression of cancer. Various studies, in this context, have highlighted the role of the nuclear factor NF-κB in the management of cellular events involving programmed cell death (apoptosis), as well as hyperplasia. This nuclear transcription factor exists in a heterodimer or di-polymer form in different cell types. The activity of the transcription factor NFκB is closely related to the TLR (Toll-like receptor), a type-I transmembrane protein belonging to a 10-membered family of proteins. This receptor acts as a vital connecting knot between the innate and adaptive arms of the body’s immune system. It recognizes several endogenous ligands and the PAMPs (Pathogen Associated Molecular Patterns). They trigger the signaling cascade that results in the release of inflammatory mediators, thereby evoking an immune response. Previous research elucidated the role of TLR in pathogenesis as well as bio-immunotherapy. The transcription factor directly binds at the MMP-9 promoter, thereby resulting in the up-regulation of the matrix metalloproteinase. In colorectal cancer, AGP abrogates TLR4/NFκB/MMP-9 signaling pathway (Zhang et al., 2017; Farooqi et al., 2020).

### Wnt/*β*-Catenin Pathway

The Wnt/*β*-catenin signaling pathway is another signaling cascade playing a vital role in human colorectal cancer (CRC) progression. Therefore, it is a powerfully potent target for CRC treatment (Koveitypour et al., 2019). 19-O-triphenylmethyl AGP (RS-PP-050), an AGP analogue plays the character of the protagonist in CRC treatment by regulating the Wnt/*β*-catenin signaling machinery. This analogue suppresses the activity of T-cell factor/lymphocyte enhancer factor (TCF/LEF), thereby resulting in down-regulation of *β*-catenin expression. It also plays a contributing role in the down-regulation of the endogenous expression of Wnt target genes. Apart from this, the AGP analogues successfully decreases the expression of the active form of *β*-catenin at the translational level and has the unique ability to function independently of GSK3*β*, a negative regulator of Wnt. Interestingly, the analogues can abrogate the nuclear translocation of *β*-catenin by blocking the Ser675 phosphorylation, thereby causing Wnt inactivation and subsequent cancer regression (Reabroi et al., 2018a).

### VEGF-VEGFR2 Signaling

Another signaling pathway contributing to tumor progression is the VEGF-VEGFR2 signaling cascade. Numerous preclinical, and clinical researches, have confirmed the presence of this factor (VEGF) at a high level in many solid tumors, including colon cancer. VEGF plays an essential role in stimulating the endothelial cells for angiogenesis, thereby resulting in cancer progression. In colon cancer, overexpression of this factor has been linked to poor prognosis and metastasis of tumor. AGP effectively binds to the ATP-binding pocket of the VEGFR2, thereby inhibiting its kinase activity. Additionally, 15-Benzylidene substituted derivatives (ADN-9) of AGP successfully, regress the growth and metastasis of tumors. AGP mainly inhibits the VEGF-mediated phosphorylation of the receptor (VEGFR) by inhibition of the mitogen-activated protein kinases (MAPKs) (Yang et al., 2017).

### TRAIL-Mediated Signaling

TRAIL is a highly efficient molecule involved in the targeted elimination of malignant cells by inducing DR4/5-mediated intracellular signaling (von Karstedt et al., 2017). AGP up-regulates TRAIL-mediated cleavage of FLIP-L protein and down-regulates XIAP levels that further help in the apoptosis process. AGP also up-regulates the expression of DR4 protein in wild-type p53 expressing cancer cells. ROS generation triggers the activation of JNK-mediated signaling, and that eventually phosphorylated p53 at 81st threonine position. This occurrence is necessary for the functional stabilization of p53 protein. AGP promotes ROS generation and subsequently stabilizes p53 (Zhou et al., 2008). Although AGP-induced TRAIL functioning significantly regresses tumor progression in several *in vivo* cancer models (Deng et al., 2019), a detailed investigation is still required to certify the immune-modulatory role of AGP on the apoptosis process.

### mTOR Signaling Pathway

AGP exerts anticancer properties by inducing autophagic cell death. It inhibits the stimulation of AKT and mTOR in several cancer cells (Liu et al., 2017) and reduces the phosphorylation of mTOR at the Ser2481 position. AGP down-regulates the protein level of RAPTOR and RICTOR, members of mTORC1 and mTORC2, respectively, (Kumar et al., 2015). It helps to prevent tumor development in mice by inhibiting NLRP3-mediated inflammation in colitis and colitis-associated cancer (Guo et al., 2014).

## Role of Andrographolide in Increasing the Efficacy of Conventional Chemotherapy for CRC

In this section, we reviewed the research findings related to the effect of AGP in colorectal cancer. Recently, the application of phytomedicines to conventional chemotherapeutic drugs has become a clinical interest. The main two reasons are their immunomodulatory effects and fewer adverse side effects (Ranjan et al., 2019). AGP has great potentiality as an anticancer phytomedicine and has been applied to treat cancer (Rajagopal et al., 2003). The anticancer properties of AGP and its functioning mechanisms have been discussed already in the previous sections. These imply that it can counter-attack the causes of CRC to a great extent. AGP, as an adjuvant with chemotherapeutic drugs, increases the therapeutic efficacy against CRC. Activation of c-MET induced signaling cascade leads to uncontrolled cell growth, proliferation, metastasis, and angiogenesis. Su et al. showed AGP enhanced 5-Fluro Uracil (5-FU)- induced anti-tumor effect in human colon cancer cell line HCT-116 by inhibiting c-MET pathway (Su et al., 2017). AGP has the potentiality to reverse the 5-FU resistance (5-FUR) in CRC by up-regulating BAX expression (Wang et al., 2016). Another research revealed high cytotoxicity when AGP co-administered with chemotherapeutic agent cisplatin in CRC. Recent researches have highlighted the efficiency of this phytomedicine in mediating both the extrinsic and intrinsic apoptotic pathways in the CRC LoVo cell line. The combinatorial therapy involving the administration of CDDP chemotherapy along with AGP increases the apoptotic rate of cancer cells which could be demonstrated by checking the changes in the transcriptional as well as translational levels of Bax and Bcl2 (Lin et al., 2014). Banerjee et al. supported this statement by their reported work that AGP stimulates ER stress and apoptosis in colon cancer cell lines (T84, HCT116, and COLO 205), that control the unwanted growth of carcinoma (Banerjee et al., 2016). A very interesting work has been done by Sharda et al. They analyzed the effect of AGP and melatonin combinatorial drug on metastatic colon cancer cell lines (T84, Colo 205, HT-29, and DLD-1) and on a metastatic patient-derived organoid model (PDOD). The result implied that this therapy induces ER stress-mediated apoptosis in metastatic CRC cell death by stimulating the IRE-1/XBP-1/CHOP signaling pathway (Sharda et al., 2021). Zhang et al. reported the anti-proliferation role of AGP on the colon cancer SW620 cell line. AGP inhibits TLR4, MyD88, NF-κB-p65, and MMP-9 signaling pathways and can be used as a promising drug to treat CRC (Zhang et al., 2017). Li et al. also reported the inhibitory role of AGP on PI3K-AKT-mTOR signaling pathway (Li et al., 2020). Henhena et al. reported that due to the antioxidant property of AGP, it down-regulated the expression and functioning of the marker genes for CRC development. AGP exhibited its efficacy as a genetic and epigenetic modulator. They also reported the chemo-preventive effect of AGP on the CRC against the carcinogen azoxymethane (Al-Henhena et al., 2014).

An analogue of AGP, 19-O-triphenylmethyl AGP (RS-PP-050) is a potent drug for CRC. Its activity has been observed on the Wnt/*β*-catenin pathway. RS-PP-050 inhibited the proliferation and survival of HT-29 CRC cells. It also induces cell cycle arrest and initiates apoptotic cell death, associated with the stimulation of PARP-1 and p53. Moreover, it has inhibitory effects on *β*-catenin transcription by down-regulating T-cell factor or lymphocyte enhancer factor (TCF/LEF) activity in cells. This analogues reduced the protein expression of the active form of *β*-catenin. Surprisingly, it dephosphorylates at Ser675 of *β*-catenin which links to the intervention of the nuclear translocation of *β*-catenin and contributes to Wnt inactivation (Reabroi et al., 2018b).

So, it is cleared that AGP can control the uncontrolled growth of colorectal polyp into carcinoma and can treat CRC by modulating the causes of CRC. Even after such promising outcomes of AGP applications on CRC cell lines, to the best of our knowledge, no significant animal model study has been done yet. The clinical trial of AGP is highly encouraging, but before that, it still requires more in-depth research (Table 1).

**TABLE 1:** Summery of the recent researches that have been done to inspect the role of Andrographolide on CRC cell lines.

Effect of andrographolide on colorectal cell lines (researches have been done so far)		
Author	CRC cell lines	Effects
Su et al. (2017)	HCT-116	Down-regulation of c-MET pathway 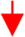
		Stimulation of 5-FU- induced anti-tumor effect 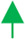
Wang et al. (2016)	HCT116/5-FUR cells	Up-regulation of Bax protein 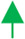
Lin et al. (2014)	CRC LoVo	Increased cytotoxicity, apoptosis, and Bax protein 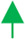
		Down-regulation of Bcl2 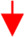
Banerjee et al. (2016)	T84, HCT116 and COLO 205	Increased ER stress and apoptosis 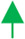
Sharda et al. (2021)	T84, Colo 205, HT-29, and DLD-1 and PDOD	Up-regulation of IRE-1/XBP-1/CHOP signaling pathway, ER stress and apoptosis 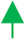
Zhang et al. (2017)	SW620	Inhibition of TLR4, MyD88, NF‐kB‐p65, and MMP‐9 signaling pathways 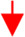
Li et al. (2020)	HCT-116	Down‐regulation of PI3K‐AKT‐mTOR signaling pathway 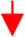
Al-Henhena et al. (2014)	CCD841 and HT29	Increased antioxident property 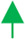
		Inhibition of CRC development genes 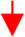
Reabroi et al. (2018b)	HT29	Inhibition of Wnt/*β*- catenin signaling pathway 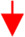
		Stimulation of Cell cycle arrest, apoptotic cell death, PARP1, p53 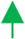

## Bioavailibity and the Methods of Application: The Drawbacks and Solutions

Alike other phytochemicals, AGP also has low aqueous solubility and poor bioavailability after oral administration. It has better solubility in the solvents, such as acetone, methanol, chloroform, and ether (Sareer et al., 2014). After oral administration of AGP, i.e., 200 mg/day, the highest concentration that was measured in human plasma was 58.62 ng/ml at 1.6 h. AGP has a half-life of 10.50 h in humans (Xu et al., 2009). For this reason, the therapeutic use of AGP is somehow restricted. It urges a necessity to develop alternative methods of application for better therapeutic results and better bioavailability. The optimum absorption of the substance is mainly depended on the properties of the solvent. These nanostructure pharmaceutical formulations containing the phytomedicine as compound delivery systems therapeutically enhance the performance of the biochemical drugs, and the functioning depends on the absorption property of the solvent (Butnariu et al., 2020). Hence, AGP has been formulated as micro- or nanoparticles for therapeutic purposes. The microparticles include polylactic-glycolic acid, alginic acid, and glucan derivatives, whereas nanoparticles include vesicles, polymeric nanoparticles, solid lipid nanoparticles, gold nanoparticles, nanocrystals, microemulsions, and nano-emulsions, and nanosuspensions (Casamonti et al., 2019). The bioavailability of AGP has been increased by 241% by the nanoparticle’s formulation than the only suspension. These formulations help to overcome the aqueous insolubility of AGP (Yang et al., 2013).

*Polymeric nanoparticles (PNPs)*: PNPs are the most common drug delivery formulation. It delivers drugs by nanospheres and nanocapsules. Synthetic and natural, both types of polymers are used to prepare PNPs. Some polymers are biodegradable in nature, and some are non-biodegradable (Bilia et al., 2017). *Polymeric micelles (PM)*: AGP is coated in a PM formulation depending on an amphiphilic triblock copolymer of D, L-lactic acid, glycolic acid, and ethylene glycol (Zhang et al., 2014). *Vesicles*: Vesicles are bi-layered colloidal vectors that can carry both hydrophilic and hydrophobic compounds. Liposomes are vesicles that have been used to load AGP to the tumor site (Bilia et al., 2016). *Solid lipid nanoparticles (SLNs)*: SLNs are spherical lipid nanoparticles of diameter 10–1,000 nm, and they can disperse in water or aqueous solution. AGP entrapped into SLNs (diameter 286.1 nm) enhanced its bioavailability and its efficacy at the tumor site (Parveen et al., 2014). *Nanoemulsions (NEs) and microemulsions (MEs):* Both NEs and MEs are characterized by high aqueous solubility and AGP delivery via these enhance its bioavailability (McClements, 2012). *Mesoporous nanoparticles (MNPs)*: MNPs are a dense framework with porous structure and large surface area that allows the attachment of different functional group for targeted the drug delivery to a particular site. *Gold nanoparticles (GNPs):* GNPs are the most advanced nanoparticles develop for biomedical usages. The targeted delivery of AGP with GNPs is promising to enhance tumor-specific cytotoxicity (Das et al., 2018). *Nanocrystals and nanosuspensions*: These two are designed for fast-dissolving and better dissolution of AGP (Ma et al., 2018).

**FIGURE 5:**
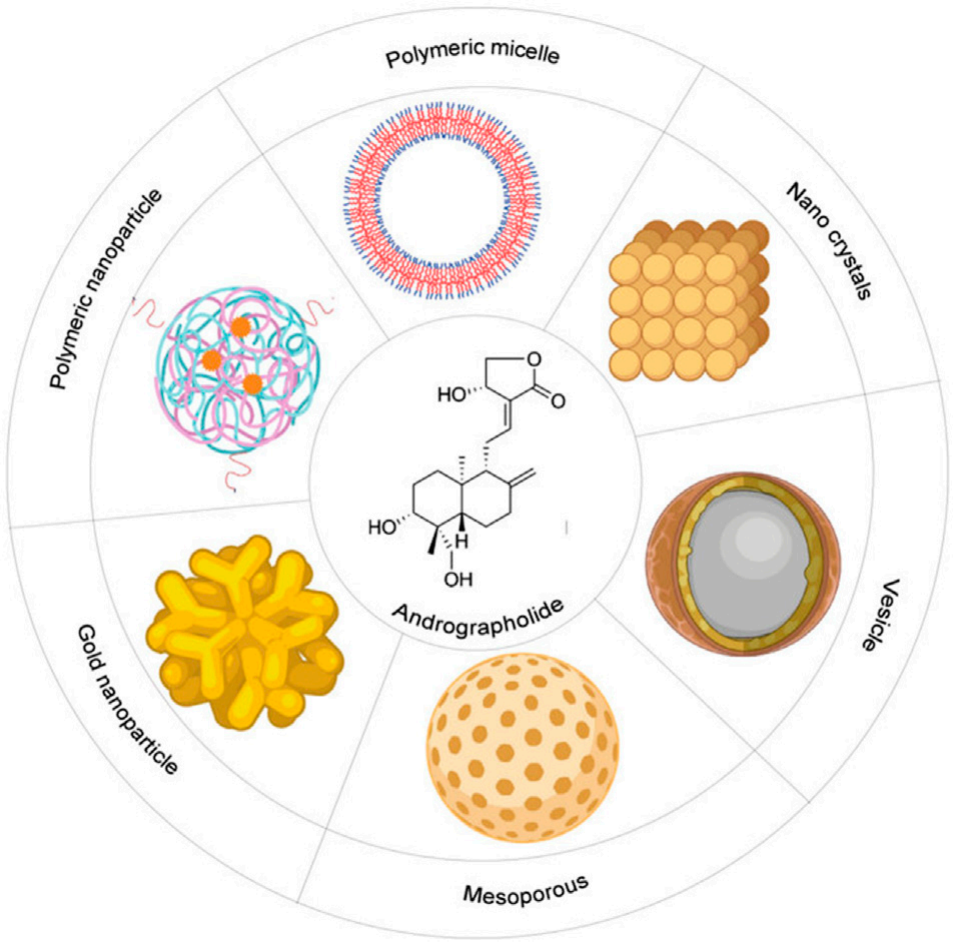
Schematic diagram of different types of nano-formulations to increase the bioavailability of the Andrographolide: It includes polymeric micelle, nano-crystals, vesicles, mesoporous, gold-nanoparticles, polymeric nanoparticles.

All these above-mentioned nanoformulations can be administrated by all methods, can control the rate and degree of drug absorption, enhance aqueous solubility, increase bioavailability, drug stability, and can be effective to overcome multidrug resistance in tumor cells (Figure 5, created by BioRender and Canvas).

## Conclusion

AGP, a widely used phytomedicine of many South-Asian countries has attracted the limelight due to its potent anti-cancer attributes. Numerous studies performed by different groups of scientists confirmed the potent, and effective neoplastic as well as immunomodulatory properties of this phytochemical.

It is now a well-corroborated certitude that an integrated and interlinked approach is essential for cancer management and its successful eradication. Thus, a compound or a multitude of compounds that can potently affect numerous tumorigenesis-related biochemical pathways globally have been of interest to cancer biologists. AGP, as an organic compound, fights against all tumor-promoting signaling molecules and pathways. To date, the performance of AGP on CRC cell lines is very satisfactory. However, most researches are still left before exhibiting it as a forthcoming chemotherapeutic agent. For instance, the precise line of action and the signaling pathways affected by this compound should be thoroughly studied. Besides, the efficacy of this compound in *in-vivo* models especially, in higher-order animals and humans should be tested so that it can be used more readily for clinical trials. Numerous naturally derived agents have entered the clinical trials and subsequently faced termination owing to the lack of efficacy, mostly due to enormous toxicity imparted by them. The screening of the compound by performing *in vitro* bioassays on several CRC cell lines has led to its selection that can be headed to pre-clinical and clinical studies.

On that basis, AGP and its semi-synthetic derivatives are interesting prospects, which can also provide a lead for novel anti-cancer drug synthesis. Taken together, this compound and its derivatives can emerge as a promising candidate to be the next new class of chemotherapeutic agents against colorectal cancer.

## Glossary

AGP: Andrographolide
AKT: Protein kinase B
AP1: Activator protein 1
APC: Antigen presenting cell
BAX: Bcl-2-associated X
BCL2: B-cell lymphoma 2
CD: Cluster of differentiation
cdc: Cell division cycle
cdk: Cyclin-dependent kinases
COX2: Cyclooxygenase-2
DRs: Death receptors
EGFR: Epidermal growth factor receptor
ER: Endoplasmic reticulum
ERK: Extracellular-signal-regulated kinase
HIF-1*α*: Hypoxia inducible factor 1*α*
IFNɣ: Interferon gamma
IL2: Interleukin 2
IRS2: Human Insulin Receptor Substrate-2
LC3-II: LC3-phosphatidylethanolamine conjugate
LPS: Lipopolysaccharide
K-RAS: Kirsten rat sarcoma virus
MAPK: Mitogen-activated protein kinases
MLH1: MutL homolog 1
MMPs: Matrix metalloproteinases
MSH2: MutS homolog 2
mTOR: Mammalian target of rapamycin
MYH: MutY DNA glycosylase
MyD88: Myeloid differentiation primary response 88
NLRP3: NLR family pyrin domain containing 3
NK cell: Natural killer cell
PAK4: p21-Activated kinase 4
PARP-1: Poly [ADP-ribose] polymerase **1**
PI3K: Phosphoinositide 3-kinase
PI3KCA: Phosphatidylinositol-4,5-bisphosphate 3-kinase catalytic subunit alpha
PTEN: Phosphatase and Tensin Homolog deleted on Chromosome 10
ROS: Reactive oxygen species
TCF/LEF: T-cell factor or lymphocyte enhancer factor
TGFBR2: Transforming Growth Factor Beta Receptor 2
TGF*β*: Tumor growth factor *β*
TLR4: Toll-like receptor 4
TP53: Tumor protein p53
TRAIL: Tumor necrosis factor-related apoptosis-inducing ligand
VEGF: Vascular endothelial growth factor
Wnt: Wingless-related integration site

## References

[B1] Abu-Ghefreh A. A. Canatan H. Ezeamuzie C. I. (2009). *In Vitro* and *In Vivo* Anti-inflammatory Effects of Andrographolide. Int. Immunopharmacol. 9, 313–318. 10.1016/j.intimp.2008.12.002 19110075

[B2] Akbar S. (2011). Andrographis Paniculata: A Review of Pharmacological Activities and Clinical Effects. Altern. Med. Rev. 16, 66–77. PMID: 21438648. 21438648

[B3] Al-Henhena N. Ying R. P. Ismail S. Najm W. Najm W. Khalifa S. A. (2014). Chemopreventive Efficacy of Andrographis Paniculata on Azoxymethane-Induced Aberrant colon Crypt Foci *In Vivo* . PLoS One 9 (11), e111118. 10.1371/journal.pone.0111118 25390042 PMC4229078

[B4] Al-Tassan N. Chmiel N. H. Maynard J. Fleming N. Livingston A. L. Williams G. T. (2002). Inherited Variants of MYH Associated with Somatic G:C-->T:A Mutations in Colorectal Tumors. Nat. Genet. 30 (2), 227–232. 10.1038/ng828 11818965

[B5] Arber N. Eagle C. J. Spicak J. Rácz I. Dite P. Hajer J. (2006). Celecoxib for the Prevention of Colorectal Adenomatous Polyps. N. Engl. J. Med. 355 (9), 885–895. 10.1056/NEJMoa061652 16943401

[B6] Banerjee A. Ahmed H. Yang P. Czinn S. J. Blanchard T. G. (2016). Endoplasmic Reticulum Stress and IRE-1 Signaling Cause Apoptosis in colon Cancer Cells in Response to Andrographolide Treatment. Oncotarget 7 (27), 41432–41444. 10.18632/oncotarget.9180 27166181 PMC5173070

[B7] Barault L. Charon-Barra C. Jooste V. de la Vega M. F. Martin L. Roignot P. (2008). Hypermethylator Phenotype in Sporadic colon Cancer: Study on a Population-Based Series of 582 Cases. Cancer Res. 68 (20), 8541–8546. 10.1158/0008-547210.1158/0008-5472.CAN-08-1171 18922929

[B8] Barber T. D. McManus K. Yuen K. W. Reis M. Parmigiani G. Shen D. (2008). Chromatid Cohesion Defects May Underlie Chromosome Instability in Human Colorectal Cancers. Proc. Natl. Acad. Sci. U S A. 105 (9), 3443–3448. 10.1073/pnas.0712384105 18299561 PMC2265152

[B9] Bera R. Ahmed S. K. Sarkar L. Sen T. Karmakar S. (2014). Pharmacokinetic Analysis and Tissue Distribution of Andrographolide in Rat by a Validated LC-MS/MS Method. Pharm. Biol. 52, 321–329. 10.3109/13880209.2013.836544 24171780

[B10] Bilia A. R. Piazzini V. Guccione C. Risaliti L. Asprea M. Capecchi G. (2017). Improving on Nature: The Role of Nanomedicine in the Development of Clinical Natural Drugs. Planta Med. 83 (5), 366–381. 10.1055/s-0043-102949 28178749

[B11] Bilia A. R. Bergonzi M. C. Guccione C. Manconi M. Fadda A. M. Sinico C. (2016). Vesicles and Micelles: Two Versatile Vectors for the Delivery of Natural Products. J. Drug Deliv. Sci. Tech. 32 (Pt B), 241–255. 10.1016/j.jddst.2015.09.007

[B12] Bos J. L. Fearon E. R. Hamilton S. R. Verlaan-de Vries M. van Boom J. H. van der Eb A. J. (1987). Prevalence of Ras Gene Mutations in Human Colorectal Cancers. Nature 327 (6120), 293–297. 10.1038/327293a0 3587348

[B13] Bronner C. E. Baker S. M. Morrison P. T. Warren G. Smith L. G. Lescoe M. K. (1994). Mutation in the DNA Mismatch Repair Gene Homologue hMLH1 Is Associated with Hereditary Non-polyposis colon Cancer. Nature 368 (6468), 258–261. 10.1038/368258a0 8145827

[B14] Butnariu M. Sarac I. Samfira I. (2020). Spectrophotometric and Chromatographic Strategies for Exploring of the Nanostructure Pharmaceutical Formulations Which Contains Testosterone Undecanoate. Sci. Rep. 10 (1), 3569. 10.1038/s41598-020-60657-4 32107451 PMC7046639

[B15] Casamonti M. Risaliti L. Vanti G. Piazzini V. Bergonzi M. C. Bilia A. R. (2019). Andrographolide Loaded in Micro- and Nano-Formulations: Improved Bioavailability, Target-Tissue Distribution, and Efficacy of the "King of Bitters". Engineering 5, 69–75. 10.1016/j.eng.2018.12.004Dua

[B16] Cha Y. I. DuBois R. N. (2007). NSAIDs and Cancer Prevention: Targets Downstream of COX-2. Annu. Rev. Med. 58, 239–252. 10.1146/annurev.med.57.121304.131253 17100552

[B17] Chakravarti R. N. Chakravarti D. (1951). Andrographolide, the Active Constituent of Andrographis Paniculata Nees; a Preliminary Communication. Ind. Med. Gaz. 86 (3), 96–97. PMID: 14860885. 14860885 PMC5191793

[B18] Chao H. P. Kuo C. D. Chiu J. H. Fu S. L. (2010). Andrographolide Exhibits Anti-invasive Activity against colon Cancer Cells via Inhibition of MMP2 Activity. Planta Med. 76 (16), 1827–1833. 10.1055/s-0030-1250039 20539971

[B19] Chen D. Song Y. Lu Y. Xue X. (2013). Synthesis and *In Vitro* Cytotoxicity of Andrographolide-19-Oic Acid Analogues as Anti-cancer Agents. Bioorg. Med. Chem. Lett. 23 (11), 3166–3169. 10.1016/j.bmcl.2013.04.010 23628335

[B20] Chen W. Feng L. Nie H. Zheng X. (2012). Andrographolide Induces Autophagic Cell Death in Human Liver Cancer Cells through Cyclophilin D-Mediated Mitochondrial Permeability Transition Pore. Carcinogenesis 33 (11), 2190–2198. 10.1093/carcin/bgs264 22869602

[B21] Chun J. Y. Tummala R. Nadiminty N. Lou W. Liu C. Yang J. (2010). Andrographolide, an Herbal Medicine, Inhibits Interleukin-6 Expression and Suppresses Prostate Cancer Cell Growth. Genes Cancer 1 (8), 868–876. 10.1177/1947601910383416 21442031 PMC3063649

[B22] Churiyah P. Pongtuluran O. B. Rofaani E. Tarwadi (2015). Antiviral and Immunostimulant Activities of Andrographis paniculata. HAYATI J. Biosciences 22, 67–72. 10.4308/hjb.22.2.67

[B23] Das S. Halder A. Mandal S. Mazumder M. A. J. Bera T. Mukherjee A. (2018). Andrographolide Engineered Gold Nanoparticle to Overcome Drug Resistant Visceral Leishmaniasis. Artif. Cell Nanomed Biotechnol 46 (Suppl. 1), 751–762. 10.1080/21691401.2018.1435549 29421940

[B24] Davoodvandi A. Sahebnasagh R. Mardanshah O. Asemi Z. Nejati M. Shahrzad M. K. (2019). Medicinal Plants as Natural Polarizers of Macrophages: Phytochemicals and Pharmacological Effects. Curr. Pharm. Des. 25 (30), 3225–3238. 10.2174/1381612825666190829154934 31465276

[B25] Deng Y. Bi R. Guo H. Yang J. Du Y. Wang C. (2019). Andrographolide Enhances TRAIL-Induced Apoptosis via P53-Mediated Death Receptors Up-Regulation and Suppression of the NF-Кb Pathway in Bladder Cancer Cells. Int. J. Biol. Sci. 15 (3), 688–700. 10.7150/ijbs.30847 PMC6367587 30745855

[B26] Dua V. K. Ojha V. P. Roy R. Joshi B. C. Valecha N. Devi C. U. (2004). Anti-malarial Activity of Some Xanthones Isolated from the Roots of Andrographis Paniculata. J. Ethnopharmacol. 95, 247–251. 10.1016/j.jep.2004.07.008 15507344

[B27] Farooqi A. A. Attar R. Sabitaliyevich U. Y. Alaaeddine N. de Sousa D. P. Xu B. (2020). The Prowess of Andrographolide as a Natural Weapon in the War against Cancer. Cancers (Basel) 12 (8), 2159. 10.3390/cancers12082159 PMC7465495 32759728

[B28] Fishel R. Lescoe M. K. Rao M. R. Copeland N. G. Jenkins N. A. Garber J. (1993). The Human Mutator Gene Homolog MSH2 and its Association with Hereditary Nonpolyposis colon Cancer. Cell. 75 (5), 1027–1038. 10.1016/0092-8674(93)90546-3 8252616

[B29] Goss K. H. Groden J. (2000). Biology of the Adenomatous Polyposis Coli Tumor Suppressor. J. Clin. Oncol. 18 (9), 1967–1979. 10.1200/JCO.2000.18.9.1967 10784639

[B30] Grady W. M. Rajput A. Myeroff L. Liu D. F. Kwon K. Willis J. (1998). Mutation of the Type II Transforming Growth Factor-Beta Receptor Is Coincident with the Transformation of Human colon Adenomas to Malignant Carcinomas. Cancer Res. 58 (14), 3101–3104. PMID: 9679977. 9679977

[B31] Guo W. Sun Y. Liu W. Wu X. Guo L. Cai P. (2014). Small Molecule-Driven Mitophagy-Mediated NLRP3 Inflammasome Inhibition Is Responsible for the Prevention of Colitis-Associated Cancer. Autophagy 10 (6), 972–985. 10.4161/auto.28374 24879148 PMC4091180

[B32] Hanahan D. Weinberg R. A. (2000). The Hallmarks of Cancer. Cell. 100, 57–70. 10.1016/j.cell.2011.02.013 10647931

[B33] Haura E. B. Turkson J. Jove R. (2005). Mechanisms of Disease: Insights into the Emerging Role of Signal Transducers and Activators of Transcription in Cancer. Nat. Clin. Pract. Oncol. 2 (6), 315–324. 10.1038/ncponc0195 16264989

[B34] Hocker H. J. Cho K. J. Chen C. Y. Rambahal N. Sagineedu S. R. Shaari K. (2013). Andrographolide Derivatives Inhibit Guanine Nucleotide Exchange and Abrogate Oncogenic Ras Function. Proc. Natl. Acad. Sci. U S A. 110 (25), 10201–10206. 10.1073/pnas.1300016110 23737504 PMC3690838

[B35] Hossain M. S. Urbi Z. Sule A. Hafizur Rahman K. M. (2014). Andrographis Paniculata (Burm. f.) Wall. Ex Nees: a Review of Ethnobotany, Phytochemistry, and Pharmacology. ScientificWorldJournal 2014, 274905. 10.1155/2014/274905 25950015 PMC4408759

[B36] Hurwitz H. Fehrenbacher L. Novotny W. Cartwright T. Hainsworth J. Heim W. (2004). Bevacizumab Plus Irinotecan, Fluorouracil, and Leucovorin for Metastatic Colorectal Cancer. N. Engl. J. Med. 350 (23), 2335–2342. 10.1056/NEJMoa032691 15175435

[B37] Iruretagoyena M. I. Sepúlveda S. E. Lezana J. P. Hermoso M. Bronfman M. Gutiérrez M. A. (2006). Inhibition of Nuclear Factor-Kappa B Enhances the Capacity of Immature Dendritic Cells to Induce Antigen-specific Tolerance in Experimental Autoimmune Encephalomyelitis. J. Pharmacol. Exp. Ther. 318 (1), 59–67. 10.1124/jpet.106.103259 16597709

[B38] Iruretagoyena M. I. Tobar J. A. González P. A. Sepúlveda S. E. Figueroa C. A. Burgos R. A. (2005). Andrographolide Interferes with T Cell Activation and Reduces Experimental Autoimmune Encephalomyelitis in the Mouse. J. Pharmacol. Exp. Ther. 312 (1), 366–372. 10.1124/jpet.104.072512 15331658

[B39] Issa J. P. (2004). CpG Island Methylator Phenotype in Cancer. Nat. Rev. Cancer 4 (12), 988–993. 10.1038/nrc1507 15573120

[B40] Jaruchotikamol A. Jarukamjorn K. Sirisangtrakul W. Sakuma T. Kawasaki Y. Nemoto N. (2007). Strong Synergistic Induction of CYP1A1 Expression by Andrographolide Plus Typical CYP1A Inducers in Mouse Hepatocytes. Toxicol. Appl. Pharmacol. 224 (2), 156–162. 10.1016/j.taap.2007.07.008 17825862

[B41] Järvinen H. J. Aarnio M. Mustonen H. Aktan-Collan K. Aaltonen L. A. Peltomäki P. (2000). Controlled 15-year Trial on Screening for Colorectal Cancer in Families with Hereditary Nonpolyposis Colorectal Cancer. Gastroenterology 118 (5), 829–834. 10.1016/s0016-5085(00)70168-5 10784581

[B42] Kasemsuk S. Sirion U. Suksen K. Piyachaturawat P. Suksamrarn A. Saeeng R. (2013). 12-Amino-andrographolide Analogues: Synthesis and Cytotoxic Activity. Arch. Pharm. Res. 36 (12), 1454–1464. 10.1007/s12272-013-0152-0 23709127

[B43] Kaufmann S. H. Earnshaw W. C. (2000). Induction of Apoptosis by Cancer Chemotherapy. Exp. Cel Res. 256 (1), 42–49. 10.1006/excr.2000.4838 10739650

[B44] Kondo Y. Issa J. P. (2004). Epigenetic Changes in Colorectal Cancer. Cancer Metastasis Rev. 23 (1-2), 29–39. 10.1023/a10.1023/a:1025806911782 15000147

[B45] Korinek V. Barker N. Morin P. J. van Wichen D. de Weger R. Kinzler K. W. (1997). Constitutive Transcriptional Activation by a Beta-Catenin-Tcf Complex in APC-/- colon Carcinoma. Science 275 (5307), 1784–1787. 10.1126/science.275.5307.1784 9065401

[B46] Koveitypour Z. Panahi F. Vakilian M. Peymani M. Seyed Forootan F. Nasr Esfahani M. H. (2019). Signaling Pathways Involved in Colorectal Cancer Progression. Cell Biosci 9, 1–14. 10.1186/s13578-019-0361-4 31827763 PMC6889432

[B47] Kumar D. Das B. Sen R. Kundu P. Manna A. Sarkar A. (2015). Andrographolide Analogue Induces Apoptosis and Autophagy Mediated Cell Death in U937 Cells by Inhibition of PI3K/Akt/mTOR Pathway. PLoS ONE 10, e0139657. 10.1371/journal.pone.0139657 26436418 PMC4593644

[B48] Kumar R. A. Sridevi K. Kumar N. V. Nanduri S. Rajagopal S. (2004). Anticancer and Immunostimulatory Compounds from Andrographis Paniculata. J. Ethnopharmacol. 92 (2-3), 291–295. 10.1016/j.jep.2004.03.004 15138014

[B49] Kumar S. Patil H. S. Sharma P. Kumar D. Dasari S. Puranik V. G. (2012). Andrographolide Inhibits Osteopontin Expression and Breast Tumor Growth through Down Regulation of PI3 kinase/Akt Signaling Pathway. Curr. Mol. Med. 12 (8), 952–966. 10.2174/156652412802480826 22804248

[B50] Lai Y. H. Yu S. L. Chen H. Y. Wang C. C. Chen H. W. Chen J. J. (2013). The HLJ1-Targeting Drug Screening Identified Chinese Herb Andrographolide that Can Suppress Tumour Growth and Invasion in Non-small-cell Lung Cancer. Carcinogenesis 34 (5), 1069–1080. 10.1093/carcin/bgt005 23306212

[B51] Lengauer C. Kinzler K. W. Vogelstein B. (1997). Genetic Instability in Colorectal Cancers. Nature 386, 623–627. 10.1038/386623a0 9121588

[B52] Li J. Cheung H. Y. Zhang Z. Chan G. K. Fong W. F. (2007). Andrographolide Induces Cell Cycle Arrest at G2/M Phase and Cell Death in HepG2 Cells via Alteration of Reactive Oxygen Species. Eur. J. Pharmacol. 568 (1-3), 31–44. 10.1016/j.ejphar.2007.04.027 17512926

[B53] Li X. Tian R. Liu L. Wang L. He D. Cao K. (2020). Andrographolide Enhanced Radiosensitivity by Downregulating Glycolysis via the Inhibition of the PI3K-Akt-mTOR Signaling Pathway in HCT116 Colorectal Cancer Cells. J. Int. Med. Res. 48 (8), 300060520946169. 10.1177/0300060520946169 32787737 PMC7427152

[B54] Li Y. Zhang P. Qiu F. Chen L. Miao C. Li J. (2012). Inactivation of PI3K/Akt Signaling Mediates Proliferation Inhibition and G2/M Phase Arrest Induced by Andrographolide in Human Glioblastoma Cells. Life Sci. 90 (25-26), 962–967. 10.1016/j.lfs.2012.04.044 22634579

[B55] Lin H. H. Shi M. D. Tseng H. C. Chen J. H. (2014). Andrographolide Sensitizes the Cytotoxicity of Human Colorectal Carcinoma Cells toward Cisplatin via Enhancing Apoptosis Pathways *In Vitro* and *In Vivo* . Toxicol. Sci. 139, 108–120. 10.1093/toxsci/kfu032 24563380

[B56] Lin H. H. Tsai C. W. Chou F. P. Wang C. J. Hsuan S. W. Wang C. K. (2011). Andrographolide Down-Regulates Hypoxia-Inducible Factor-1α in Human Non-small Cell Lung Cancer A549 Cells. Toxicol. Appl. Pharmacol. 250 (3), 336–345. 10.1016/j.taap.2010.11.014 21134392

[B57] Liu S. H. Lin C. H. Liang F. P. Chen P. F. Kuo C. D. Alam M. M. (2014). Andrographolide Downregulates the V-Src and Bcr-Abl Oncoproteins and Induces Hsp90 Cleavage in the ROS-dependent Suppression of Cancer Malignancy. Biochem. Pharmacol. 87 (2), 229–242. 10.1016/j.bcp.2013.10.014 24161787

[B58] Liu Y. Zhang Y. Zou J. Yan L. Yu X. Lu P. (2017). Andrographolide Induces Autophagic Cell Death and Inhibits Invasion and Metastasis of Human Osteosarcoma Cells in an Autophagy-dependent Manner. Cell Physiol Biochem 44 (4), 1396–1410. 10.1159/000485536 29197865

[B59] Ma Y. Yang Y. Xie J. Xu J. Yue P. Yang M. (2018). Novel Nanocrystal-Based Solid Dispersion with High Drug Loading, Enhanced Dissolution, and Bioavailability of Andrographolide. Int. J. Nanomedicine 13, 3763–3779. 10.2147/IJN.S164228 29988798 PMC6030943

[B60] Madav S. Tripathi H. C. Mishra S. K. (1995). Analgesic, Antipyretic and Antiulcerogenic Effects of Andrographolide. Indian J. Pharm. Sci. 57, 121.

[B61] Markowitz S. D. Bertagnolli M. M. (2009). Molecular Origins of Cancer: Molecular Basis of Colorectal Cancer. N. Engl. J. Med. 361 (25), 2449–2460. 10.1056/NEJMra0804588 20018966 PMC2843693

[B62] Matsuda T. Kuroyanagi M. Sugiyama S. Umehara K. Ueno A. Nishi K. (1994). Cell Differentiation-Inducing Diterpenes from Andrographis Paniculata Nees. Chem. Pharm. Bull. (Tokyo) 42, 1216–1225. 10.1248/cpb.42.1216 8069972

[B63] McClements D. J. (2012). Nanoemulsions versus Microemulsions: Terminology, Differences, and Similarities. Soft Matter 8 (6), 1719–1729. 10.1039/C2SM06903B

[B64] Mishra S. K. Sangwan N. S. Sangwan R. S. (2007). Andrographis Paniculata (Kalmegh): a Review. Pharmacognosy Rev. 1 (2), 283–298. License: CC BY-NC-SA 4.0.

[B65] Mishra S. K. Tripathi S. Shukla A. Oh S. H. Kim H. M. (2015). Andrographolide and Analogues in Cancer Prevention. Front. Biosci. 7, 292–304. 10.2741/E732 25553378

[B66] Naik S. R. Hule A. (2009). Evaluation of Immunomodulatory Activity of an Extract of Andrographolides from Andographis Paniculata. Planta Med. 75 (8), 785–791. 10.1055/s-0029-1185398 19263340

[B67] Nateewattana J. Dutta S. Reabroi S. Saeeng R. Kasemsook S. Chairoungdua A. (2014). Induction of Apoptosis in Cholangiocarcinoma by an Andrographolide Analogue Is Mediated through Topoisomerase II Alpha Inhibition. Eur. J. Pharmacol. 723, 148–155. 10.1016/j.ejphar.2013.12.002 24360936

[B68] Nateewattana J. Saeeng R. Kasemsook S. Suksen K. Dutta S. Jariyawat S. (2013). Inhibition of Topoisomerase II α Activity and Induction of Apoptosis in Mammalian Cells by Semi-synthetic Andrographolide Analogues. Invest. New Drugs 31 (2), 320–332. 10.1007/s10637-012-9868-9 22899371

[B69] Newman D. J. Cragg G. M. (2012). Natural Products as Sources of New Drugs over the 30 Years from 1981 to 2010. J. Nat. Prod. 75 (3), 311–335. 10.1021/np200906s 22316239 PMC3721181

[B70] Nguyen H. T. Duong H. Q. (2018). The Molecular Characteristics of Colorectal Cancer: Implications for Diagnosis and Therapy. Oncol. Lett. 16 (1), 9–18. 10.3892/ol.2018.8679 29928381 PMC6006272

[B71] Pan C. W. Yang S. X. Pan Z. Z. Zheng B. Wang J. Z. Lu G. R. (2017). Andrographolide Ameliorates D-Galactosamine/lipopolysaccharide-Induced Acute Liver Injury by Activating Nrf2 Signaling Pathway. Oncotarget 8 (25), 41202–41210. 10.18632/oncotarget.17149 28465482 PMC5522263

[B72] Parveen R. Ahmad F. J. Iqbal Z. Samim M. Ahmad S. (2014). Solid Lipid Nanoparticles of Anticancer Drug Andrographolide: Formulation, *In Vitro* and *In Vivo* Studies. Drug Dev. Ind. Pharm. 40 (9), 1206–1212. 10.3109/03639045.2013.810636 23826860

[B73] Pratheeshkumar P. Kuttan G. (2011). Andrographolide Inhibits Human Umbilical Vein Endothelial Cell Invasion and Migration by Regulating MMP-2 and MMP-9 during Angiogenesis. J. Environ. Pathol. Toxicol. Oncol. 30 (1), 33–41. 10.1615/jenvironpatholtoxicoloncol.v30.i1.40 21609314

[B74] Qin L. H. Kong L. Shi G. J. Wang Z. T. Ge B. X. (2006). Andrographolide Inhibits the Production of TNF-Alpha and Interleukin-12 in Lipopolysaccharide-Stimulated Macrophages: Role of Mitogen-Activated Protein Kinases. Biol. Pharm. Bull. 29 (2), 220–224. 10.1248/bpb.29.220 16462022

[B75] Rajagopal S. Kumar R. A. Deevi D. S. Satyanarayana C. Rajagopalan R. (2003). Andrographolide, a Potential Cancer Therapeutic Agent Isolated from Andrographis Paniculata. J. Exp. Ther. Oncol. 3 (3), 147–158. 10.1046/j.1359-4117.2003.01090.x 14641821

[B76] Rajagopalan H. Bardelli A. Lengauer C. Kinzler K. W. Vogelstein B. Velculescu V. E. (2002). Tumorigenesis: RAF/RAS Oncogenes and Mismatch-Repair Status. Nature 418 (6901), 934. 10.1038/418934a 12198537

[B77] Ranjan A. Ramachandran S. Gupta N. Kaushik I. Wright S. Srivastava S. (2019). Role of Phytochemicals in Cancer Prevention. Int. J. Mol. Sci. 20 (20), 4981. 10.3390/ijms20204981 PMC6834187 31600949

[B78] Reabroi S. Chairoungdua A. Saeeng R. Kasemsuk T. Saengsawang W. Zhu W. (2018a). A Silyl Andrographolide Analogue Suppresses Wnt/β-Catenin Signaling Pathway in colon Cancer. Biomed. Pharmacother. 101, 414–421. 10.1016/j.biopha.2018.02.119 29501763

[B79] Reabroi S. Saeeng R. Boonmuen N. Kasemsuk T. Saengsawang W. Suksen K. (2018b). The Anti-cancer Activity of an Andrographolide Analogue Functions through a GSK-3β-independent Wnt/β-Catenin Signaling Pathway in Colorectal Cancer Cells. Sci. Rep. 8, 7924. 10.1038/s41598-018-26278-8 29784906 PMC5962551

[B80] Roy P. Das S. Mondal A. Chatterji U. Mukherjee A. (2012). Nanoparticle Engineering Enhances Anticancer Efficacy of Andrographolide in MCF-7 Cells and Mice Bearing EAC. Curr. Pharm. Biotechnol. 13 (15), 2669–2681. 10.2174/138920112804724855 23072387

[B81] Saltz L. B. Meropol N. J. LoehrerSr P. J. Needle M. N. Kopit J. Mayer R. J. (2004). Phase II Trial of Cetuximab in Patients with Refractory Colorectal Cancer that Expresses the Epidermal Growth Factor Receptor. J. Clin. Oncol. 22 (7), 1201–1208. 10.1200/JCO.2004.10.182 14993230

[B82] Samuels Y. Wang Z. Bardelli A. Silliman N. Ptak J. Szabo S. (2004). High Frequency of Mutations of the PIK3CA Gene in Human Cancers. Science 304 (5670), 554. 10.1126/science.1096502 15016963

[B83] Sareer O. Ahmad S. Umar S. (2014). Andrographis Paniculata: a Critical Appraisal of Extraction, Isolation and Quantification of Andrographolide and Other Active Constituents. Nat. Prod. Res. 28 (23), 2081–2101. 10.1080/14786419.2014.924004 24912126

[B84] Seoane J. Gomis R. R. (2017). TGF-β Family Signaling in Tumor Suppression and Cancer Progression. Cold Spring Harb. Perspect. Biol. 9 (12), a022277. 10.1101/cshperspect PMC5710110 28246180

[B85] Shapouri-Moghaddam A. Mohammadian S. Vazini H. Taghadosi M. Esmaeili S. A. Mardani F. (2018). Macrophage Plasticity, Polarization, and Function in Health and Disease. J. Cel Physiol. 233 (9), 6425–6440. 10.1002/jcp.26429 PMC13482560 29319160

[B86] Sharda N. Ikuse T. Hill E. Garcia S. Czinn S. J. Bafford A. (2021). Impact of Andrographolide and Melatonin Combinatorial Drug Therapy on Metastatic Colon Cancer Cells and Organoids. Clin. Med. Insights Oncol. 15, 11795549211012672. 10.1177/11795549211012672 34158803 PMC8182223

[B87] Sheeja K. Kuttan G. (2007b). Activation of Cytotoxic T Lymphocyte Responses and Attenuation of Tumor Growth *In Vivo* by Andrographis Paniculata Extract and Andrographolide. Immunopharmacol Immunotoxicol 29 (1), 81–93. 10.1080/08923970701282726 17464769

[B88] Sheeja K. Kuttan G. (2007a). Modulation of Natural Killer Cell Activity, Antibody-dependent Cellular Cytotoxicity, and Antibody-dependent Complement-Mediated Cytotoxicity by Andrographolide in normal and Ehrlich Ascites Carcinoma-Bearing Mice. Integr. Cancer Ther. 6 (1), 66–73. 10.1177/1534735406298975 17351028

[B89] Sheeja K. Kuttan G. (2006). Protective Effect of Andrographis Paniculata and Andrographolide on Cyclophosphamide-Induced Urothelial Toxicity. Integr. Cancer Ther. 5 (3), 244–251. 10.1177/1534735406291984 16880430

[B90] Shi M. D. Lin H. H. Lee Y. C. Chao J. K. Lin R. A. Chen J. H. (2008). Inhibition of Cell-Cycle Progression in Human Colorectal Carcinoma Lovo Cells by Andrographolide. Chem. Biol. Interact 174 (3), 201–210. 10.1016/j.cbi.2008.06.006 18619950

[B91] Siegel R. L. Miller K. D. Goding Sauer A. Fedewa S. A. Butterly L. F. Anderson J. C. (2020). Colorectal Cancer Statistics, 2020. CA Cancer J. Clin. 70 (3), 145–164. 10.3322/caac.21601 32133645

[B92] Sirion U. Kasemsook S. Suksen K. Piyachaturawat P. Suksamrarn A. Saeeng R. (2012). New Substituted C-19-Andrographolide Analogues with Potent Cytotoxic Activities. Bioorg. Med. Chem. Lett. 22, 49–52. 10.1016/j.bmcl.2011.11.085 22154665

[B93] Su M. Qin B. Liu F. Chen Y. Zhang R. (2017). Andrographolide Enhanced 5-Fluorouracil-Induced Antitumor Effect in Colorectal Cancer via Inhibition of C-MET Pathway. Drug Des. Devel Ther. 11, 3333–3341. 10.2147/DDDT.S140354 PMC5703152 29200829

[B94] Sukumari-Ramesh S. Bentley J. N. Laird M. D. Singh N. Vender J. R. Dhandapani K. M. (2011). Dietary Phytochemicals Induce P53- and Caspase-independent Cell Death in Human Neuroblastoma Cells. Int. J. Dev. Neurosci. 29 (7), 701–710. 10.1016/j.ijdevneu.2011.06.002 21704149 PMC3818802

[B95] Tan M. L. Tan H. K. Oon C. E. Kuroyanagi M. Muhammad T. S. (2012). Identification of Genes Involved in the Regulation of 14-Deoxy-11,12-Didehydroandrographolide-Induced Toxicity in T-47D Mammary Cells. Food Chem. Toxicol. 50, 431–444. 10.1016/j.fct.2011.11.001 22101062

[B96] Toyota M. Ahuja N. Ohe-Toyota M. Herman J. G. Baylin S. B. Issa J. P. (1999). CpG Island Methylator Phenotype in Colorectal Cancer. Proc. Natl. Acad. Sci. U S A. 96 (15), 8681–8686. 10.1073/pnas.96.15.8681 10411935 PMC17576

[B97] Varma A. Padh H. Shrivastava N. (2011). Andrographolide: A New Plant-Derived Antineoplastic Entity on Horizon. Evid. Based Complement. Alternat Med. 2011, 815390. 10.1093/ecam/nep135 19752167 PMC3139959

[B98] von Karstedt S. Montinaro A. Walczak H. (2017). Exploring the TRAILs Less Travelled: TRAIL in Cancer Biology and Therapy. Nat. Rev. Cancer. 17 (6), 352–366. 10.1038/nrc.2017.28 28536452

[B99] Wang W. Guo W. Li L. Fu Z. Liu W. Gao J. (2016). Andrographolide Reversed 5-FU Resistance in Human Colorectal Cancer by Elevating BAX Expression. Biochem. Pharmacol. 121, 8–17. 10.1016/j.bcp.2016.09.024 27693317

[B100] Wang W. Wang J. Dong S. F. Liu C. H. Italiani P. Sun S. H. (2010). Immunomodulatory Activity of Andrographolide on Macrophage Activation and Specific Antibody Response. Acta Pharmacol. Sin. 31 (2), 191–201. 10.1038/aps.2009.205 20139902 PMC4002847

[B101] Wiart C. Kumar K. Yusof M. Y. Hamimah H. Fauzi Z. M. Sulaiman M. (2005). Antiviral Properties of Ent-Labdene Diterpenes of Andrographis Paniculata Nees, Inhibitors of Herpes Simplex Virus Type 1. Phytother Res. 19 (12), 1069–1070. 10.1002/ptr.1765 16372376

[B102] Wolpin B. M. Mayer R. J. (2008). Systemic Treatment of Colorectal Cancer. Gastroenterology 134 (5), 1296–1310. 10.1053/j.gastro.2008.02.098 18471507 PMC2528832

[B103] Wu Y. Zhou B. P. (2009). Inflammation: a Driving Force Speeds Cancer Metastasis. Cell Cycle 8 (20), 3267–3273. 10.4161/cc.8.20.9699 19770594 PMC3702728

[B104] Xie Y. H. Chen Y. X. Fang J. Y. (2020). Comprehensive Review of Targeted Therapy for Colorectal Cancer. Signal. Transduct Target. Ther. 5 (1), 22. 10.1038/s41392-020-0116-z 32296018 PMC7082344

[B105] Xu L. Xiao D. W. Lou S. Zou J. J. Zhu Y. B. Fan H. W. (2009). A Simple and Sensitive HPLC-ESI-MS/MS Method for the Determination of Andrographolide in Human Plasma. J. Chromatogr. B Analyt Technol. Biomed. Life Sci. 877 (5-6), 502–506. 10.1016/j.jchromb.2008.12.065 19158000

[B106] Xu Y. Tang D. Wang J. Wei H. Gao J. (2019). Neuroprotection of Andrographolide against Microglia-Mediated Inflammatory Injury and Oxidative Damage in PC12 Neurons. Neurochem. Res. 44 (11), 2619–2630. 10.1007/s11064-019-02883-5 31562575

[B107] Yang L. Wu D. Luo K. Wu S. Wu P. (2009). Andrographolide Enhances 5-Fluorouracil-Induced Apoptosis via Caspase-8-dependent Mitochondrial Pathway Involving P53 Participation in Hepatocellular Carcinoma (SMMC-7721) Cells. Cancer Lett. 276 (2), 180–188. 10.1016/j.canlet.2008.11.015 19097688

[B108] Yang T. Sheng H. H. Feng N. P. Wei H. Wang Z. T. Wang C. H. (2013). Preparation of Andrographolide-Loaded Solid Lipid Nanoparticles and Their *In Vitro* and *In Vivo* Evaluations: Characteristics, Release, Absorption, Transports, Pharmacokinetics, and Antihyperlipidemic Activity. J. Pharm. Sci. 102, 4414–4425. 10.1002/jps.23758 24166599

[B109] Yang W. Zhao J. Wang Y. Xu H. Wu Z. Hu Y. (2017). *In Vivo* inhibitory Activity of Andrographolide Derivative ADN-9 against Liver Cancer and its Mechanisms Involved in Inhibition of Tumor Angiogenesis. Toxicol. Appl. Pharmacol. 327, 1–12. 10.1016/j.taap.2017.04.022 28438631

[B110] Zhang J. Li Y. Gao W. Repka M. A. Wang Y. Chen M. (2014). Andrographolide-loaded PLGA-PEG-PLGA Micelles to Improve its Bioavailability and Anticancer Efficacy. Expert Opin. Drug Deliv. 11 (9), 1367–1380. 10.1517/17425247.2014.924503 24935153

[B111] Zhang R. Zhao J. Xu J. Jiao D. X. Wang J. Gong Z. Q. (2017). Andrographolide Suppresses Proliferation of Human colon Cancer SW620 Cells through the TLR4/NF-Κb/mmp-9 Signaling Pathway. Oncol. Lett. 14, 4305–4310. 10.3892/ol.2017.6669 28943944 PMC5604146

[B112] Zhou J. Hu S. E. Tan S. H. Cao R. Chen Y. Xia D. (2012). Andrographolide Sensitizes Cisplatin-Induced Apoptosis via Suppression of Autophagosome-Lysosome Fusion in Human Cancer Cells. Autophagy 8 (3), 338–349. 10.4161/auto.18721 22302005

[B113] Zhou J. Lu G. D. Ong C. S. Ong C. N. Shen H. M. (2008). Andrographolide Sensitizes Cancer Cells to TRAIL-Induced Apoptosis via P53-Mediated Death Receptor 4 Up-Regulation. Mol. Cancer Ther. 7 (7), 2170–2180. 10.1158/1535-7163.MCT-08-0071 18645026

[B114] Zhou J. Ong C. N. Hur G. M. Shen H. M. (2010). Inhibition of the JAK-STAT3 Pathway by Andrographolide Enhances Chemosensitivity of Cancer Cells to Doxorubicin. Biochem. Pharmacol. 79 (9), 1242–1250. 10.1016/j.bcp.2009.12.014 20026083

[B115] Zhu Y. Y. Yu G. Zhang Y. Xu Z. Wang Y. Q. Yan G. R. (2013). A Novel Andrographolide Derivative AL-1 Exerts its Cytotoxicity on K562 Cells through a ROS-dependent Mechanism. Proteomics 13 (1), 169–178. 10.1002/pmic.201200273 23161516

